# Novel localization of formin mDia2: importin β-mediated delivery to and retention at the cytoplasmic side of the nuclear envelope

**DOI:** 10.1242/bio.013649

**Published:** 2015-10-30

**Authors:** Xiaowei Shao, Keiko Kawauchi, G. V. Shivashankar, Alexander D. Bershadsky

**Affiliations:** 1 Mechanobiology Institute, National University of Singapore, 117411, Singapore; 2 Frontiers of Innovative Research in Science and Technology, Konan University, Kobe 650-0047, Japan; 3 Department of Biological Sciences, National University of Singapore, 117543, Singapore; 4 FIRC Institute of Molecular Oncology, Milan 20139, Italy; 5 Department of Molecular Cell Biology, Weizmann Institute of Science, Rehovot 76100, Israel

**Keywords:** Formin mDia2, Nuclear rim, Importin β

## Abstract

The formin family proteins are important regulators of actin polymerization that are involved in many cellular processes. However, little is known about their specific cellular localizations. Here, we show that Diaphanous-related formin-3 (mDia2) localizes to the cytoplasmic side of the nuclear envelope. This localization of mDia2 to the nuclear rim required the presence of a nuclear localization signal (NLS) sequence at the mDia2 N-terminal. Consistent with this result, super-resolution images demonstrated that at the nuclear rim, mDia2 co-localized with the nuclear pore complexes and a nuclear transport receptor, importin β. Furthermore, an interaction between mDia2 and importin β was detected by immunoprecipitation, and silencing of importin β was shown to attenuate accumulation of mDia2 to the nuclear rim. We have shown previously that Ca^2+^ entry leads to the assembly of perinuclear actin rim in an inverted formin 2 (INF2) dependent manner. mDia2, however, was not involved in this process since abolishing its localization at the nuclear rim by silencing of importin β had no effect on actin assembly at the nuclear rim triggered by Ca^2+^ stimulation.

## Abstract (precis)

**Summary:** Formin mDia2 accumulates to the cytoplasmic side of the nuclear envelope and co-localizes with the nuclear pore complexes in an importin β-dependent manner.

## INTRODUCTION

The formin family proteins have emerged as important regulators of actin assembly and cytoskeletal remodeling. They are involved in various functional regulations of the global actin network as well as specialized actin-based structures (reviewed in Campellone and Welch, 2010; Chesarone et al., 2010). There is some evidence that in addition to their cytoplasmic functions, some formins could be associated with the nucleus. In particular, formin FHOD1 interacts with nesprin, an essential component of the LINC complex (linker of nucleoskeleton and cytoskeleton) (Crisp et al., 2006), and mediates formation of transmembrane actin-associated nuclear (TAN) lines and nuclear movement (Kutscheidt et al., 2014). Another formin, inverted formin-2 (INF2), localizes to endoplasmic reticulum (ER) and the nuclear rim, where it mediates a unique force- and Ca^2+^-induced transient actin polymerization (Shao et al., 2015).

A member of the Diaphanous-related formins, mDia2, which is best known for inducing filopodia and lamellipodia formation (Pellegrin and Mellor, 2005; Yang et al., 2007; Harris et al., 2010), has been found to shuttle between the nucleus and the cytoplasm (Miki et al., 2009). A short N-terminal sequence [16-39 amino acids (aa)] of mDia2 has been identified as a functional nuclear localization signal (NLS) that can be recognized by the nuclear transport receptor importin α (Miki et al., 2009). In the presence of a nuclear export inhibitor, Leptomycin B (LMB), mDia2 accumulates within the nucleus (Miki et al., 2009). Although these experiments indicate that mDia2 could localize inside the nucleus, and mDia2 has been implicated in catalyzing nuclear actin polymerization (Baarlink et al., 2013), the presence of intra-nuclear mDia2 in cells not treated with LMB has not been observed.

Using confocal imaging, we detected an accumulation of mDia2 at the outer nuclear membrane of cells in the absence of LMB treatment. Super-resolution microscopy revealed that mDia2 co-localized with the nuclear pore complexes (NPC) as well as a nuclear transport receptor, importin β. This localization at the nuclear rim was dependent on the NLS sequence of mDia2 and the interaction of mDia2 with importin β. This study provides an alternative explanation of the link between mDia2 and nuclear transport machinery, whereby mDia2 is targeted to the nuclear rim by importin β and may participate in the regulation of cellular functions at the perinuclear region.

## RESULTS

### Accumulation of mDia2 at the cytoplasmic side of the nuclear envelope

**Fig. 1.**
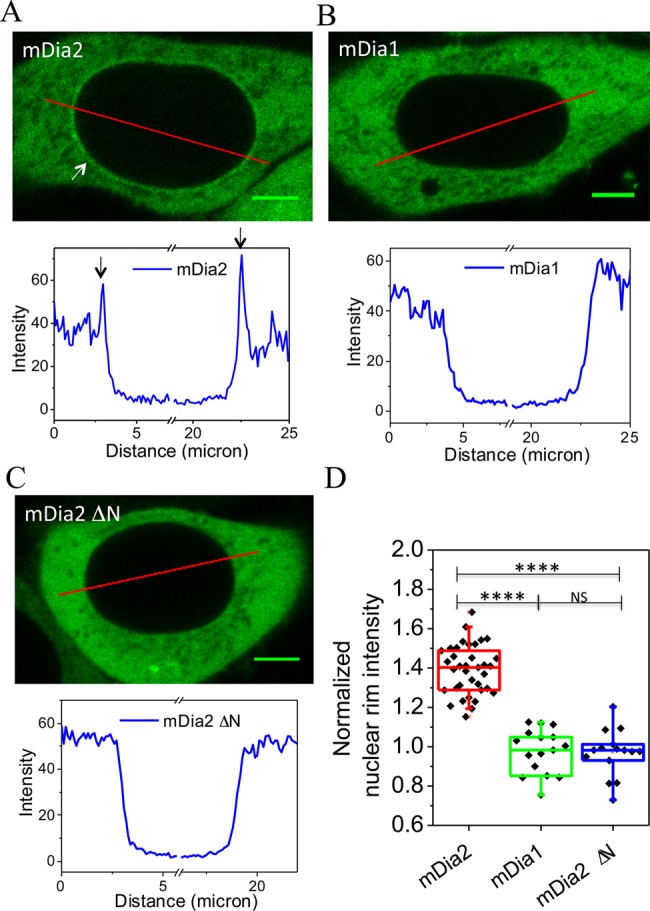
**Accumulation of mDia2 to the nuclear rim.** (A-C) Fluorescence images of HeLa JW cells expressing EGFP-mDia2 (A), EGFP-mDia1 (B), EGFP-mDia2 ΔN (C) and profiles of fluorescence intensity along the red line in the corresponding images (graphs). Arrows indicate localization of mDia2 at the nuclear rim. Scale bars: 5 μm. (D) Quantification of nuclear rim intensity from cells expressing EGFP-mDia2, EGFP-mDia1 and EGFP-mDia2 ΔN. ‘Normalized nuclear rim intensity’ was defined as the ratio between the intensity at the nuclear rim and in the proximity of the cytoplasm. ****P<0.0001; NS, *P*>0.05; two-tailed unpaired Student's *t*-test.

In order to study the localization of mDia2, HeLa JW cells were transfected with EGFP-fused full-length mDia2 and examined under confocal microscopy. Formin mDia1 was chosen as a control to mDia2. Consistently with previous studies, both mDia1 and mDia2 were diffusely localized throughout the cytoplasm with extremely low intra-nuclear signal (Pellegrin and Mellor, 2005; Beli et al., 2008; Miki et al., 2009; Gorelik et al., 2011). Here, we found that EGFP-mDia2, and not mDia1, showed an enhanced localization at the nuclear rim (Fig. 1A,B, top row). Fluorescence profiles indicating line intensity in the images show peaks at the nuclear rim for mDia2 but not mDia1 (Fig. 1A,B, bottom row). Since it has been reported that mDia2 shuttles between the nucleus and the cytoplasm (Miki et al., 2009), we further tested whether the NLS sequence of mDia2 played a role in its localization to the nuclear rim. A mutant of mDia2 lacking the complete NLS, mDia2 ΔN (33-1171 aa), did not demonstrate nuclear shuttling (Fig. S1A) and was unable to localize to the nuclear rim (Fig. 1C), suggesting that the NLS of mDia2 was necessary for this localization. Quantitatively, the intensity of full-length EGFP-mDia2 at the nuclear rim (1.41±0.16) normalized by its intensity in the cytoplasm in the proximity of the rim, was approximately 40% higher than the analogous parameters for mDia1 (0.98±0.11) and mDia2 ΔN (1.03±0.28) (Fig. 1D). Importantly, immunostaining revealed that endogenous mDia2 localized to the nuclear rim similarly to EGFP-mDia2 (Fig. S1B). Compared to endogenous mDia1, the intensity of endogenous mDia2 at nuclear rim was significantly higher (Fig. S1C). These results suggest that mDia2 localizes to the nuclear rim depending on its N-terminal NLS sequence.

**Fig. 2.**
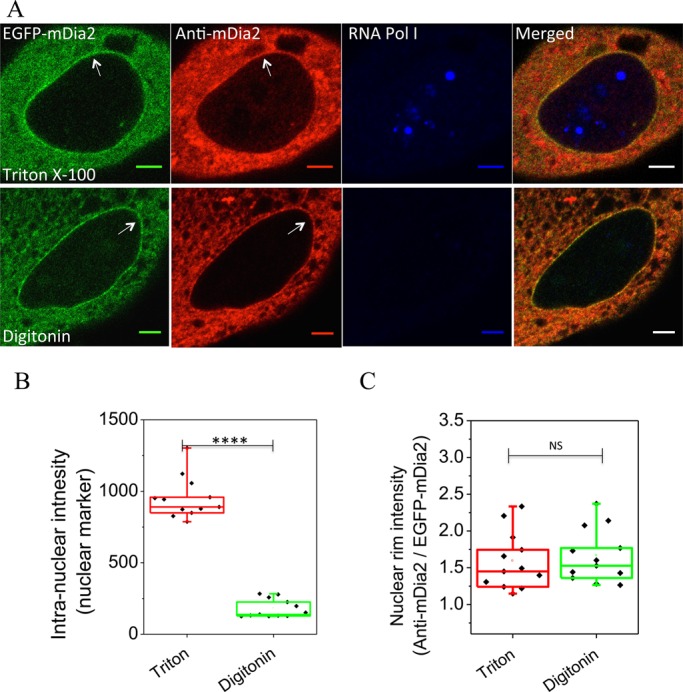
**mDia2 localization at the cytoplasmic side of the nuclear envelope.** (A) Immunostaining of mDia2 (red) and an intra-nuclear protein PRAF1, a component of RNA polymerase I (RNA Pol I, blue), in EGFP-mDia2 (green) expressing cells permeabilized by either Triton X-100 (upper) or digitonin (lower). Superimposed images are shown on the right. Note that digitonin does not permeabilize nuclear membranes. Arrows indicate enrichment of mDia2 at the nuclear rim. Scale bars: 5 μm. (B) Quantification on intensity of PRAF1 inside the nucleus in cells permeabilized by either Triton X-100 or digitonin. (C) Quantification on intensity of mDia2 immunostaining (normalized by that of EGFP-mDia2) at the nuclear rim in the same cells as quantified in (B). ****P<0.0001; NS, *P*>0.05; two-tailed unpaired Student's *t*-test.

To determine whether mDia2 localizes to the outer or inner side of the nuclear envelope, we performed immunostaining using different methods of permeabilization. While Triton X-100 permeabilizes all membrane structures, a low concentration of digitonin only permeabilizes the plasma membrane while still keeping the nuclear membranes intact (Adam et al., 1990). Therefore, mDia2 accumulation at the nuclear rim should be visible via immunofluorescence only if it localizes at the cytoplasmic side of the nuclear envelope. Immunostaining using an antibody against mDia2 revealed its enrichment at the nuclear rim in EGFP-mDia2 expressing cells permeabilized by either Triton X-100 or digitonin (Fig. 2A, arrows). Unlike mDia2, immunostaining for the intra-nuclear protein RNA polymerase I showed a significant difference depending on permeabilization procedures. RNA polymerase I could be visualized after Triton X-100 but not digitonin permeabilization (Fig. 2A, blue channel; Fig. 2B, compared to Fig. 2C), similarly to another intra-nuclear protein, heterochromatin protein-1α (data not shown). These results indicate that mDia2 localizes at the cytoplasmic side of the nuclear envelope.

Fluorescence correlation spectroscopy (FCS) was used to compare the dynamics of mDia2 within the cytoplasm and at the nuclear rim. In cells transfected with EGFP-mDia2, FCS measurements were taken in the same cell, within the bulk of the cytoplasm and the ventral part of the nuclear rim, as indicated in Fig. S2A. The autocorrelation function (ACF) curves obtained from FCS were fitted with a 3-dimensional anomalous diffusion model (Weiss et al., 2004). Direct outputs of the fitting are diffusion time (τ_D_), the average time taken a fluorophore to diffuse through the illuminated volume, and the anomalous factor (α) that indicates the degree of the anomalous sub-diffusion (Foo et al., 2012). Here, the diffusion time τ_D_ of mDia2 was shown to be longer at the nuclear rim compared to within the cytoplasm (Fig. S2B). Conversely, α was reduced at the nuclear rim, indicating that diffusion of mDia2 is more restricted when in close proximity to the nucleus (Fig. S2C). At both the cytoplasm and the nuclear rim, τ_D_ and α showed significant anti-correlation (i.e. when τ_D_ is bigger, α tends to be smaller) (Fig. S2D). Altogether, the FCS data show that mDia2 is less diffusive at the nuclear rim than in the cytoplasm. This suggests that mDia2 may be trapped by some interaction with proteins associated to the nuclear envelope.

### Possible partners of mDia2 at the nuclear rim

**Fig. 3.**
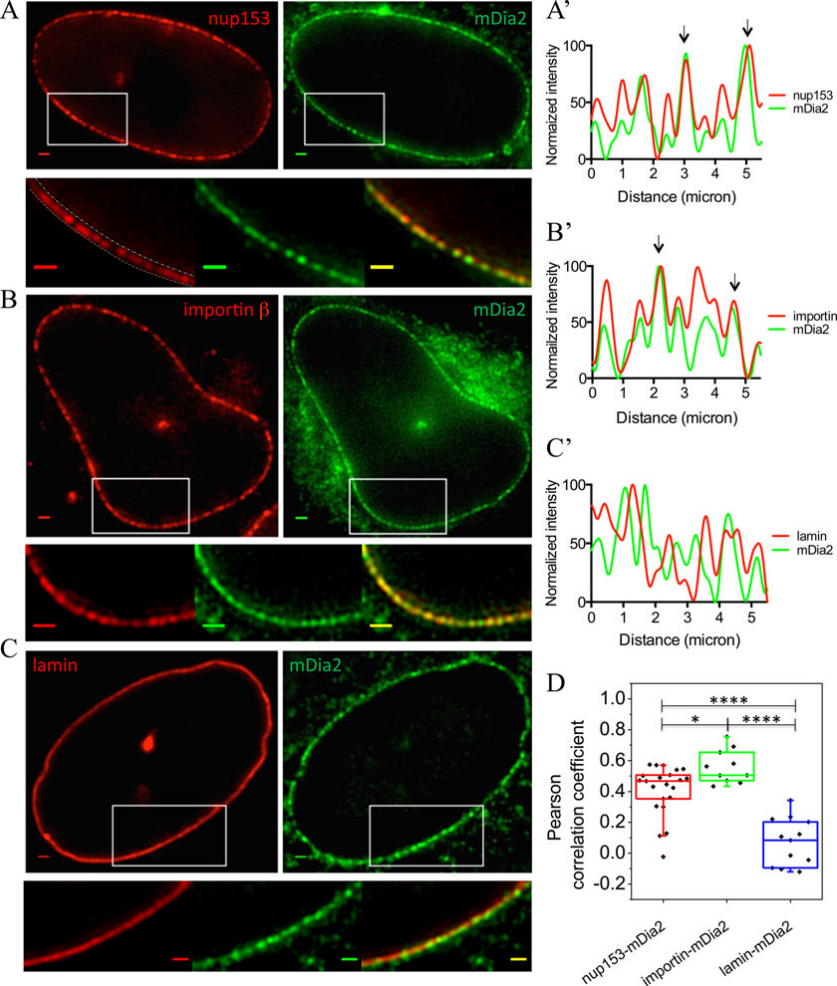
**Co-localization of mDia2 with the nuclear pore complexes and importin β at the nuclear rim.** (A-C) Upper row: reconstructed SIM images of EGFP-mDia2 (green) and (A) nup153, (B) importin β and (C) lamin B1 (red). Lower row: high-magnification images of the individual channels and superimposed image from the regions indicated by the white rectangles. Scale bars: 1 μm. The dashed lines on the high-magnification red channel image of A indicate the border of the nuclear rim. Quantification of fluorescence intensity along the nuclear rim was done by drawing a line with a width of ∼20 pixels on the rim, and the average intensity along the width was taken for each point on the line. (A′-C′) Normalized fluorescence intensity along the nuclear rim in cells labeled with mDia2 (green curve) together with nup153, importin β or lamin B1 (red curve). Co-localizations are indicated by arrows. (D) Pearson correlation coefficient between the fluorescence intensity of the two corresponding channels shown in A-C along the nuclear rim. ****P<0.0001; *P<0.05; two-tailed unpaired Student's *t*-test.

Structured illumination microscopy (SIM) (Gustafsson et al., 2008) was employed to examine the localization of mDia2 at the super-resolution level. After image reconstruction, discontinuous patches of mDia2 distributed along the nuclear border were revealed (Fig. 3A-C, green channel; Fig. S3A-C). As the localization pattern of mDia2 appeared similar to that of the nuclear pore complexes, we proceeded to immunostain for NPC elements as well as factors involved in nuclear transport machinery. An NPC component, Nup153, and the nuclear import receptor, importin β, were therefore labeled by immunofluorescence in cells expressing EGFP-mDia2. As a control, the nuclear lamina, a dense fibrillar network underlying the inner nuclear membrane, was also labeled by mCherry-lamin B1 in EGFP-mDia2 transfected cells. Reconstructed SIM images show that mDia2 co-localizes with both Nup153 and importin β at the nuclear rim (Fig. 3A,B). However, mDia2 does not co-localize with the nuclear lamina (Fig. 3C), consistent with the result that it is localized to the outer nuclear membrane as observed previously. The intensity profiles of mDia2, together with either Nup153, importin β, or lamin B1, are presented in Fig. 3A′-C′ (arrows indicate co-localizations). These profiles were obtained by first drawing a line with a width of approximately 20 pixels along the nuclear border on the red channel image, as illustrated in Fig. 3A (lower image), and then taking the average value along the width of the line for each point of the curve. To analyze the level of co-localization, the Pearson correlation coefficient (PCC) was calculated between the fluorescence intensity profiles of mDia2 and either Nup153, importin β, or lamin B1 along the nuclear border. Quantitatively, the PCC between importin β and mDia2 (0.56±0.11, mean±s.d.), and between Nup153 and mDia2 (0.41±0.16), were both significantly higher than between lamin B1 and mDia2 (0.08±0.15) (Fig. 3D). These results suggest that importin β and nuclear transport machinery can be involved in localizing mDia2 to the nuclear rim.

mDia2 has been shown to be activated by Rac1 GTPase and plays a role during enucleation of mammalian erythroblasts (Ji et al., 2008; Lammers et al., 2008). We observed, in agreement with (Kraynov et al., 2000), localization of active Rac1 (Q61L) at the nuclear rim in a considerable number of cells (Fig. S4A). To find out whether Rac1 activity is involved in the accumulation of mDia2 at the nuclear rim, cells expressing EGFP-mDia2 were treated with Rac1 inhibitor NSC 23766. However, this treatment did not remove mDia2 from nuclear rim (Fig. S4B,C), suggesting that Rac1 activity is not required for mDia2 localization at the nuclear rim.

### The essential role of importin β in localizing mDia2 to the nuclear rim

**Fig. 4.**
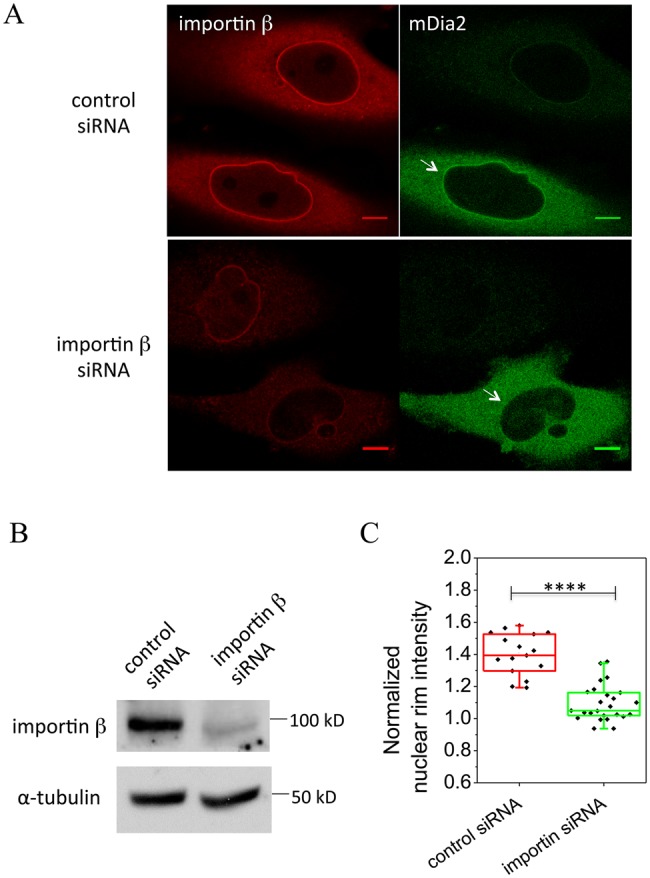
**Importin β knockdown affects mDia2 localization to the nuclear rim.** (A) Immunostaining of importin β (red) in cells transfected with control or importin β siRNAs together with EGFP-mDia2 (green). Arrows indicate mDia2 localization without or with importin β knockdown. In each image, the upper cells in the field of observation were not transfected by EGFP-mDia2. Scale bars: 10 μm. (B) Immunoblots of importin β in control and knockdown cells. α-tubulin was used as an internal control. (C) Quantification of nuclear rim intensity from cells expressing EGFP-mDia2 in control and importin β siRNA treated cells. ‘Normalized nuclear rim intensity’ was defined as a ratio between mDia2 intensity at the nuclear rim and in the proximity of the cytoplasm. ****P<0.0001; two-tailed unpaired Student's *t*-test.

To understand the involvement of importin β in the localization of mDia2 to the nuclear rim, the effect of importin β knockdown was examined. Human importin β siRNAs (SMARTpool, GE Dharmacon) were transfected into HeLa JW cells together with EGFP-mDia2. Immunostaining of importin β showed cytoplasmic localization with enhanced concentration at the nuclear rim, consistently with previous studies (Ciciarello et al., 2004; Ma et al., 2007; Wu et al., 2009). Knockdown of importin β significantly reduced its immunofluorescence under the same microscopy setting (Fig. 4A). Western blot analysis showed that the expression of importin β was reduced by ∼70% after siRNA treatment (Fig. 4B). The decrease in the level of importin β was largely associated with a reduction of mDia2 intensity at the nuclear rim (Fig. 4A). In importin β silenced cells, the ratio between mDia2 intensity at the nuclear rim and in the cytoplasm significantly dropped to 1.10±0.02, compared to 1.41±0.03 from cells transfected with non-targeting control siRNAs (Fig. 4C).

**Fig. 5.**
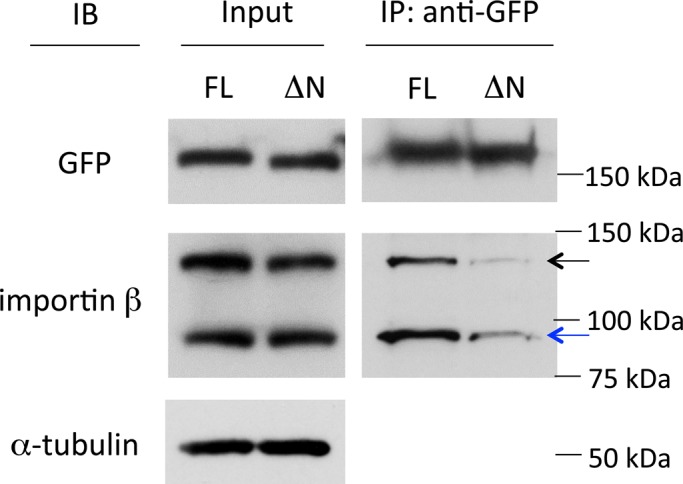
**Interaction between mDia2 and importin β detected by immunoprecipitation (IP) assay.** Cell lysate co-transfected with EGFP-mDia2 [full-length (FL) or 33-1171 aa (ΔN)] and mCherry-importin β were incubated with GFP antibody and protein G Sepharose beads. Portions of the input (whole cell lysate) and bound proteins from IP were separated by SDS-PAGE followed by immunoblotting (IB) using antibodies against GFP, importin β and α-tubulin. Black arrow indicates exogenous mCherry-importin β, and blue arrow indicates endogenous importin β. Full-length mDia2 pulled down both endogenous importin β and mCherry-importin β at a significantly higher level than mDia2 with an N-terminal truncation. α-tubulin was used as an internal control.

Although it was previously demonstrated that the nuclear import of mDia2 was dependent on importin β, an *in vitro* binding assay using purified proteins only revealed an interaction between mDia2 and importin α, the transport adaptor of importin β (Miki et al., 2009). To examine the interaction of mDia2 and importin β, an immunoprecipitation assay was performed. In cell lysate, full-length EGFP-mDia2 immunoprecipitated with both exogenous mCherry-importin β and endogenous importin β (Fig. 5). mDia2 ΔN mutant, which did not localize to the nuclear rim (Fig. 1C), displayed significantly weaker binding to importin β (Fig. 5). These results suggest that mDia2 associates with importin β via its N-terminal NLS sequence, and this interaction is essential for mDia2 localization to the nuclear rim.

We have previously shown that an intracellular Ca^2+^ burst induces assembly of perinuclear actin (Shao et al., 2015). This assembly was shown to depend on the formin INF2, which is localized to the ER and enriched at the nuclear rim. Since mDia2 also localizes to the nuclear rim, we decided to check whether mDia2 was involved in Ca^2+^-induced perinuclear actin assembly. However, displacement of mDia2 from the nuclear rim after silencing of importin β did not lead to a significant reduction in perinuclear actin assembly upon the treatment with the calcium ionophore A23187 (Fig. S4D-F). This result indicates that importin β, as well as mDia2 localization at the nuclear rim, is not required for Ca^2+^-induced perinuclear actin assembly. Moreover, using a constitutively active (CA) mDia2 construct (411-1171 aa), in which the N-terminus of mDia2 (containing endogenous NLS) was substituted with a ‘classical’ NLS sequence, localization of active mDia2 to the nuclear rim and even the nuclear interior was enhanced (Fig. S4G). However, cells expressing such NLS-CA mDia2 showed no alteration in the level of perinuclear F-actin or observable intra-nuclear F-actin, as compared to those expressing CA mDia2 without the NLS (Fig. S4G-I). Thus, localization of mDia2 to the nuclear rim is neither necessary nor sufficient for the activation of actin polymerization at this location.

## DISCUSSION

In this study, a novel localization of formin mDia2 to the nuclear rim was described. Although previously the nuclear shuttling of mDia2 has been described (Miki et al., 2009), the direct evidence for its nuclear and perinuclear localization in the absence of Leptomycin B was missing. We demonstrate here that mDia2 localized to the external surface of the nuclear envelope. This localization was detected not only for exogenous EGFP-mDia2 but also for endogenous mDia2, and therefore cannot be explained by mDia2 over-expression. Further, using super-resolution structured illumination microscopy, we found that at the nuclear rim, mDia2 distribution was similar to that of nuclear pore complexes, and was also closely associated with the nuclear transport machinery. Importin β co-localizes with mDia2 at the nuclear rim, and this nuclear rim localization of mDia2 depends on its interaction with importin β via the NLS sequence of mDia2.

The role of importin α has been indicated to be necessary for the nuclear import of mDia2 previously (Miki et al., 2009). Interaction of importin α and mDia2 fragment (16-39 aa), which was shown to be a functional NLS, has also been demonstrated by *in vitro* binding assay in that study. Thus, it is possible that mDia2 interacts with importin β via the adaptor protein importin α, as many other cargo proteins. The accumulation of mDia2 at the nuclear pores could be, therefore, a result of a ‘traffic jam’ as it travels from cytoplasm to the nucleus. An analogous traffic jam accumulation was suggested for cargoes of importin β that enriched at the nuclear rim due to limited transport efficiency (Yang and Musser, 2006). The nuclear transport of mDia2 is probably strictly regulated. Therefore, delivery of mDia2 to the vicinity of the nuclear pores may be insufficient for the nuclear import, and some additional factors/signals are necessary to permit its entry.

However, it is possible that accumulation of mDia2 at the external part of the nuclear rim mediated by importin β is not just a consequence of its unsuccessful delivery to the nucleus, but is required for a specific biological function. Many cargoes transported by importin β are essentially spindle assembly factors that are spatially regulated by RanGTP concentration gradient during mitosis (Kalab and Heald, 2008). Several lines of evidence have indicated that mDia2 is tightly associated with mitosis. It plays a key role in cytokinesis, the final phase of mitosis, by inducing formation of the contractile actomyosin ring (Watanabe et al., 2008, 2010). The expression level of mDia2 is precisely regulated in the cell cycle, increasing during S phase and mitosis, and declining at the end of mitosis (DeWard and Alberts, 2009). Further, knockdown of mDia2 affects spindle formation in mouse oocytes (Kim et al., 2015). Given the important roles of mDia2 in the progression of mitosis, we speculate that the delivery and accumulation of mDia2 to the nuclear rim could be an important step in the preparation of its mitotic function.

Finally, the major function of formins known so far is promotion of actin polymerization. In some situations, actin is polymerized around the nuclear rim and we have shown recently that such polymerization induced by Ca^2+^ entry is mediated by formin INF2. We investigated here whether mDia2 was also involved in this process, and did not find evidence for this function. It could be, however, that mDia2 still participates in the formation of perinuclear actin rim triggered by other signals. In addition, mDia2 could mediate interactions of nuclear envelope with the surrounding actin network or other cytoskeletal components such as the microtubule network, since mDia2 interaction with microtubules was previously documented (Bartolini et al., 2008; Gaillard et al., 2011). Thus, we have shown here that mDia2 formin can be delivered to the external side of the nuclear envelope via its interaction with importin β and accumulates at this location. The functional role of such localization provides an interesting avenue for future studies.

## MATERIALS AND METHODS

### Cell culture, plasmids and transfection

HeLa JW cells and HEK 293T cells were cultured in Dulbecco's Modified Eagle's Medium supplemented with 10% (v/v) heat-inactivated fetal bovine serum and 1% penicillin-streptomycin at 37°C in a 5% CO_2_ incubator. All cell culture reagents were purchased from Gibco Life Technologies. For inhibition of nuclear export, cells were treated with 10-20 nM Leptomycin B (A.G. Scientific) for 1 h. For inhibition of Rac1 activity, cells were treated with 50 μM NSC 23766 (Sigma) for 16 h. For Ca^2+^ stimulation, cells were treated with 10 μM calcium ionophore A23187 (Sigma) in growth medium for 5 min.

The plasmids encoding EGFP-fused full-length mDia2, mDia2 ΔN (33-1171 aa), constitutively active (CA) mDia2 (411-1171 aa), NLS-CA mDia2, and full-length mDia1 were kind gifts from Dr S. Narumiya (Faculty of Medicine, Kyoto University, Japan) and were described previously (Watanabe et al., 1999, 2008; Miki et al., 2009). Full-length lamin B1 was sub-cloned into a pmCherry-C1 vector from EGFP-lamin B1 (Bhattacharya et al., 2009). mCherry-importin β was sub-cloned from EGFP-importin β, a kind gift from Dr P. Lavia (Institute of Molecular Biology and Pathology, CNR National Research Council, Italy), by swapping EGFP into mCherry between the *Age*I and *Not*I sites.

DNA plasmid transfection was performed using either Lipofectamine (Life Technologies), Fugene HD (Roche) or Gene Juice (Merck Millipore), according to manufacturer's instructions. All transfected cells were incubated for 24-48 h before experiments. For siRNA transfection, 100 nM of human importin β siRNAs (SMARTpool, GE Dharmacon) or non-targeting control siRNAs (SMARTpool, GE Dharmacon) were co-transfected with EGFP-mDia2 plasmid into HeLa JW cells using DharmaFECT Duo Transfection Reagent (GE Dharmacon). siRNA transfected cells were incubated for 48-72 h before experiments.

### Immunofluorescence and antibodies

Prior to immunofluorescence staining, cells were fixed with 4% paraformaldehyde (PFA) for 15-20 min at room temperature. Permeabilization was performed by incubation with 0.2% Triton X-100 (Sigma) in PBS for 10 min at room temperature. For mild permeabilization to keep nuclear membranes intact, cells were treated with 0.003% digitonin (Sigma) in PBS for 15 min on ice (Adam et al., 1990). Permeabilized cells were then blocked with 3% BSA (Sigma) in PBS for 1 h, followed by primary antibody and secondary antibody staining for 1 h each. F-actin was labeled by Alexa-563-conjugated phalloidin (Life Technologies) for 20-30 min after permeabilization.

Primary antibodies used for immunofluorescence included the following: polyclonal rabbit anti-mDia2 (gift from Dr A. S. Alberts, Van Andel Research Institute, Michigan, USA, described in Wallar et al., 2007); polyclonal mouse anti-PRAF1 (Abcam); monoclonal mouse anti-Nup153 (gift from Dr B. Burke, Institute of Medical Biology, A*STAR, Singapore); monoclonal mouse anti-importin β (Abcam); monoclonal mouse anti-mDia1 (BD Transduction Laboratories). Secondary antibodies used for immunofluorescence were: Alexa Fluor 405, 488, 568 and 647-conjugated anti-mouse or anti-rabbit immunoglobulin antibodies (Life Technologies).

### Immunoblotting and immunoprecipitation assay

For immunoblotting, transfected cells were lysed in 1× SDS loading buffer. Samples were then sonicated and subjected to SDS-PAGE. Proteins were transferred to PVDF membranes at 100 V for 1.5 h and blocked for 30 min with 5% low-fat milk in TBS-T before the addition of primary antibodies. Primary antibodies were either incubated for 1 h at room temperature or overnight at 4°C. After washing with TBS-T, the membrane was incubated with HRP-conjugated secondary antibodies for 20 min. Bound antibodies were detected by HRP Chemiluminescent reagent (Thermo Scientific).

For immunoprecipitation (IP), HEK 293T cells were scraped using a rubber policeman in cold PBS 36 h after transfection with EGFP-mDia2 expression vector together with mCherry-importin β expression vector, and lysed in RIPA buffer (50 mM Tris-HCl, 150 mM NaCl, 10 mM EDTA, 1% Triton X-100, pH 8.0) containing protease inhibitor cocktail (Roche) and phosphatase inhibitors. The IP assay was made up of cell lysate containing 2 mg proteins, 4-5 µg GFP antibody (Medical & Biological Laboratory), 20 µl protein G sepharose beads (GE Healthcare) and topped up with lysis buffer to a final volume of 500-600 µl. The mixture was gently rotated in a 1.5 ml Eppendorf tube for 2 h at 4°C. After incubation, the unbound fraction was removed and the beads were washed three times with RIPA buffer. Protein samples were separated using 6% or 7.5% polyacrylamide Tris-Glycine gels and proteins of interest were detected by immunoblotting. Antibodies used for immunoblotting and IP were: monoclonal mouse anti-α-tubulin (Sigma); monoclonal mouse anti-importin β (Abcam); polyclonal rabbit anti-GFP (Medical & Biological Laboratory); HRP-conjugated anti-mouse or anti-rabbit secondary antibodies (Bio-Rad).

### Microscopy and data analysis

Confocal image acquisition was done on Nikon A1R and Carl Zeiss LSM710 confocal microscope systems using 60× or 100× oil immersion objectives (NA 1.4). Structured illumination microscopy was performed on a Nikon N-SIM system using 3D-SIM mode and a 100× oil objective lens (NA 1.49). Calibration and channel alignment of SIM were carried out using 100 nm multi-fluorescence beads. Imaging reconstruction was done using built-in NIS-Elements Software on the Nikon microscope. Confocal images and reconstructed SIM images were analyzed using Fiji (ImageJ, National Institutes of Health) image processing package.

For co-localization analysis, the Pearson correlation coefficient (PCC, r) was calculated between the intensity profiles of two proteins along the nuclear borderline. PCC computation was performed using Excel (Microsoft) and Prism 6 (GraphPad). Statistical analysis was done using Prism 6 and Origin Software. Data were examined by F-test for variances, followed by a two-tailed Student's *t*-test. Data is presented as a box plot showing the median, 25% and 75% quartiles, with the whiskers representing the 5% and 95% boundaries.

### Fluorescence correlation spectroscopy (FCS)

The principles and model fitting of FCS are described in (Foo et al., 2012). In short, the intensity fluctuations of a fluorescence signal F(t) was measured with time in FCS measurements. FCS allows us to estimate the value of diffusion time (τ_D_), which is the average time taken by a fluorophore to diffuse through the illuminated volume, and the anomalous factor (α), indicating the difference between the real, observed diffusion and ideal free diffusion (Weiss et al., 2004; Bhattacharya et al., 2006). A value of α<1 indicates sub-diffusion, with smaller α values representing more hindered diffusion; while α>1 indicates super-diffusion, in which active cellular transport may play a role.

The FCS experiments were performed using the ConfoCor 3 module on a Zeiss LSM710 confocal microscope with avalanche photodiode (Perkin Elmer) and 40× water objective lens (NA 1.2, Carl Zeiss). The pinhole size was kept at 50 µm for the 488 nm laser. System calibration was done using Atto488 (Sigma) solution on a 0.175 mm glass coverslip (Iwaki). The diffusion time of Atto488 at 25°C was measured to be ∼25 µs after fitting with a 3-dimensional free diffusion model. FCS measurements were performed on live cells expressing EGFP-mDia2 at 24 h after transfection. Before FCS experiments, the culture medium was changed to Opti-MEM without phenol red (Life Technologies) to minimize background. Cells were kept at 37°C during the measurements. Three locations were chosen for FCS measurements in each cell at both the nuclear rim and the cytoplasm, respectively. Data were collected for 10-s interval over 10 runs at each point of measurement. For measurements at the nuclear rim, the focus was fine-tuned until the ventral side of nuclear rim was reached. All experimental autocorrelation function (ACF) curves were fitted using a 3-dimensional anomalous diffusion model. ACF curve fitting was accomplished using Igor Pro software with a build-in FCS data processing program provided by Dr T. Wohland (Department of Biological Sciences, National University of Singapore, Singapore).
